# Interaction between macrophages and ferroptosis

**DOI:** 10.1038/s41419-022-04775-z

**Published:** 2022-04-16

**Authors:** Yan Yang, Yu Wang, Lin Guo, Wen Gao, Ting-Li Tang, Miao Yan

**Affiliations:** 1 Department of Pharmacy, Second Xiangya Hospital, Central South University, Changsha, China; 2 Xiangya School of Medicine, Central South University, Changsha, China

**Keywords:** Cell death and immune response, Cytokines, Cancer immunotherapy

## Abstract

**Abstract:**

Ferroptosis, a newly discovered iron-dependent cell death pathway, is characterized by lipid peroxidation and GSH depletion mediated by iron metabolism and is morphologically, biologically and genetically different from other programmed cell deaths. Besides, ferroptosis is usually found accompanied by inflammatory reactions. So far, it has been found participating in the development of many kinds of diseases. Macrophages are a group of immune cells that widely exist in our body for host defense and play an important role in tissue homeostasis by mediating inflammation and regulating iron, lipid and amino acid metabolisms through their unique functions like phagocytosis and efferocytosis, cytokines secretion and ROS production under different polarization. According to these common points in ferroptosis characteristics and macrophages functions, it’s obvious that there must be relationship between macrophages and ferroptosis. Therefore, our review aims at revealing the interaction between macrophages and ferroptosis concerning three metabolisms and integrating the application of certain relationship in curing diseases, mostly cancer. Finally, we also provide inspirations for further studies in therapy for some diseases by targeting certain resident macrophages in distinct tissues to regulate ferroptosis.

**Facts:**

- Ferroptosis is considered as a newly discovered form characterized by its nonapoptotic and iron-dependent lipid hydroperoxide, concerning iron, lipid and amino acid metabolisms.
- Ferroptosis has been widely found playing a crucial part in various diseases, including hepatic diseases, neurological diseases, cancer, etc.
- Macrophages are phagocytic immune cells, widely existing and owning various functions such as phagocytosis and efferocytosis, cytokines secretion and ROS production.
- Macrophages are proved to participate in mediating metabolisms and initiating immune reactions to maintain balance in our body.
- Recent studies try to treat cancer by altering macrophages’ polarization which damages tumor microenvironment and induces ferroptosis of cancer cells.

**Open questions:**

- How do macrophages regulate ferroptosis of other tissue cells specifically?
- Can we use the interaction between macrophages and ferroptosis in treating diseases other than cancer?
- What can we do to treat diseases related to ferroptosis by targeting macrophages?
- Is the use of the relationship between macrophages and ferroptosis more effective than other therapies when treating diseases?

## Introduction

Ferroptosis is a newly discovered cell programmed death that is found in various diseases, such as renal diseases, hepatic diseases, neurological diseases, etc. [1–4]. The manifestation of ferroptosis is different from other cell death like apoptosis or necrosis for its iron-dependent lipid peroxidization [5]. Fenton reaction between Fe^2+^ and Fe^3+^ contributes a lot to the production of ROS. The transcription and expression of iron metabolic genes such as transferrin (TRF), ferroportin (FPN), FTH and FTL are closely related with occurrence of ferroptosis [6–12]. In addition to iron metabolism, lipid and amino acid metabolism play a part in ferroptosis as well. In order to deal with the damage caused by ROS, GPX4 and GSH are responsible for regulating ROS level via Nrf2 pathway [13–16]. As GPX4 or GSH deplete, ROS accumulation in cells can directly lead to lipid peroxidation. Besides, overactivation of ACSL4 and LOXs can also convert PUFAs into lipid peroxide. All processes above make up the causes of ferroptosis [17–26]. After study the characteristics and consequences of ferroptosis, iron overload and some of the substances released from ferroptotic cells are proved to affect the polarization and recruitment of macrophages [27–36].

Macrophages, a significant group of immune cells in the body, were firstly discovered for their clearance of foreign substances. They usually exist in two distinct phenotypes, the classically activated (M1 type) macrophages or the alternatively activated (M2 type) macrophages. After further studied, macrophages are classified into different categories according their origination or functions under various circumstances [37–40]. The inflammatory factors like IL-6, IL-1β and TNF-α that initiate inflammation response are mostly secreted by M1 type macrophages, and they influence the activity of enzymes in iron, lipid and amino acid metabolisms [40–57]. What’s more, hemophagocytosis of macrophages shows their tight relationship with the iron metabolism and production for other tissue cells [27–31, 58]. Besides, ROS produced by macrophages have effects on the state of both tissue cells and macrophages themselves by activating various pathways concerning iron, lipid and amino acid metabolisms [32–36, 59–62].

Due to the similarity in macrophages’ functions and the manifestations of ferroptotic cells, there must have been interactions between the occurrence of ferroptosis and the state of macrophages. So far, the relationship between ferroptosis and macrophages have been applied to studying the therapy for kinds of diseases, especially cancer [63–70]. Therefore, it’s meaningful to integrate the key points of the two sides in three metabolisms and provide inspirations for further studies.

## The overview of ferroptosis

**Fig. 1:**
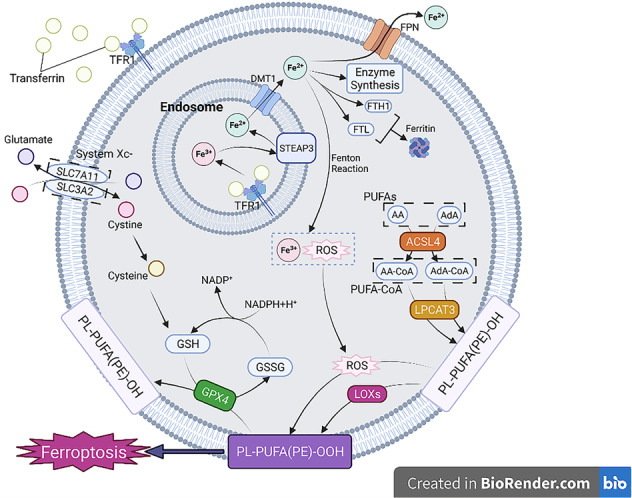
The overview of ferroptosis. The process of ferroptosis is associated with three main metabolisms (iron, lipid and amino acid metabolisms). Cells intake iron into endosome in the form of Fe^3+^ through binding between transferrin and TFR1. Fe^3+^ can be reduced to Fe^2+^ by STEAP3, which is then transported into cytosol by DMT1. Most of Fe^2+^ can be released by FPN, used for enzyme synthesis or stored in ferritin. The rest of Fe^2+^ may change into Fe^3+^ and ROS by Fenton Reaction. ROS accumulation will cause ferroptosis for it make PL-PUFA(PE)-OH oxidized into PL-PUFA(PE)-OOH. System Xc-, composed of SLC7A11 and SLC3A2, acts as a transporter responsible for releasing glutamate and intaking cysteine, which can be further transformed in to cysteine that is an important component of GSH. GSH can deoxidize PL-PUFA(PE)-OOH back into PL-PUFA(PE)-OH with the help of GPX4, which protects cells from ferroptosis. PUFAs, especially AA and AdA, are changed into PUFA-CoAs which is modified by LPCAT3 in order to be integrated into cell membrane. Catalyzed by LOXs, PL-PUFA(PE)-OH changes into PL-PUFA(PE)-OOH, leading to ferroptosis.

Ferroptosis is considered as a newly discovered form of Regulated Cell Death in 2012 by Dixon that is characterized by its nonapoptotic and iron-dependent lipid hydroperoxide [5]. Researchers have proved that various diseases are related to the ferroptosis, such as cardiovascular diseases, neurological diseases, hepatic toxicity, kidney injury, etc. [1–4]. Ferroptosis is mainly driven by peroxidation of membrane phospholipids (PLs) in enzymatic and nonenzymatic ways. The overload of iron and the inactivation of GPX4 in ferroptotic cells implies the relationship with the metabolism of iron, lipid and amino acid (Fig. 1).

### Related metabolism pathway

#### Iron metabolism

Iron is transported from extracellular environment into intracellular space through transferrin receptor 1 located on the cell membrane in the form of ferric ion (Fe^3+^) [6], which is reduced to ferrous ion (Fe^2+^) by ferric reductases such as STEAP3 in endosome [7] and then released into cytosol by divalent metal transporter 1 [8], participating in the synthesis of iron-dependent enzymes. The rest part of Fe^2+^ is generally stored in the labile iron pool or ferritin composed of ferritin heavy chain 1 and ferritin light chain (FTL) [9]. Ferroportin1 (FPN) is the identified iron exporter in cells [10]. The loss of FPN is an important contributor to iron overload and the development of ferroptosis [11]. Toxicity of iron ions comes from the Fenton Reaction between Fe^2+^ and Fe^3+^, producing reactive oxygen species (ROS) [12] which can impair lipids, proteins and DNA and cause ferroptosis.

#### Lipid metabolism

Lipid oxidization and reduction generally maintain a dynamic balance in normal cells, while excessive lipid oxide is found in ferroptotic cells, due to external factors or the disorder in related gene expression. Evidence has proved that ferroptosis is triggered by peroxidation of PLs [17]. Ferroptosis shows obvious lipid peroxidation stress and damage in cell membrane. Polyunsaturated fatty acids (PUFAs) are believed to contribute to the form of lipid peroxide and the occurrence of ferroptosis [18]. Among all kinds of PUFAs. Moreover, studies suggested that arachidonic acid and adrenic acid (AdA) are the significant contributors to the occurrence of ferroptosis. Esterified by Acyl-CoA synthetase long-chain family member-4, PUFAs transform into PUFA-CoA which can be modified by lysophosphatidylcholine acyltransferase-3 in order to be integrated into the cell membrane [19, 20, 25]. The PL-PUFA(PE)-OH are further oxidized into lipid hydroperoxides (PL-PUFA(PE)-OOH) either catalyzed by lipoxygenases (LOXs) [21] or ROS produced by Fenton reaction. Besides, 4-Hydroxynonenal (4-HNE), a major product of lipid peroxidation, is reported to induce the generation of ROS, which will aggravate the lipid peroxidation and ferroptosis, so 4-HNE can be a marker of ferroptosis as well [22–24, 26].

#### Amino acid metabolism

One of the manifestations of ferroptosis is the inactivation of glutathione peroxidase 4 (GPX4) and the depletion of GSH, which is the most important antioxidized pathway to repair the damage in cell membrane caused by lipid hydroperoxides and clean the ROS. GPX4 and System Xc- are two crucial regulators of ferroptosis. System Xc- (cystine/glutamate antiporter) acts as an important antioxidant protein encoded by SLC7A11 and SLC3A2, providing cystine that is the raw material for the synthesis of GSH. Experiments have proved that suppressing expression of System Xc- can make cells vulnerable to the induction of ferroptosis [13, 14]. GSH, a glutathione peroxidase, converts reduced glutathione (GSH) into oxidized glutathione (GSSG) to detoxify the hydroperoxide group (−OOH) of fatty acids [15]. Inhibiting GPX4 directly by Honokiol is able to induce ferroptosis without affecting the function of System Xc- [16].

## Background of macrophage

Macrophages are phagocytic immune cells, existing in various tissue and playing an important part in immune system. The evaluation of macrophages’ role is quite sophisticated. Though macrophages ingest harmful particles or microorganisms to maintain host defense, cytokines or ROS they produce, generally used to attack foreign substances, may also damage normal cells and cause chronic diseases.

### Origination of macrophage

People have believed that macrophages are all come from monocytes derived from bone marrow until a special group of macrophages is found in certain tissue. According to studies conducted in different ways, these macrophages are proved to have their own origination and characteristics. And therefore, macrophages are mainly divided into two types: 1. resident macrophages that are located in tissues/organs at a relatively stable rate for tissue defense and homeostasis. Evidence embodies that tissue-resident macrophages originate from Tie^2+^ (also known as Tek) cellular pathway that produces Csf1r^+^ erythro-myeloid progenitors. 2. recruited or bone marrow derived macrophages which come from adult hematopoietic stem cells (HSCs) [37]. Though resident macrophages can self-renew independent of HSCs, they are similar to recruited macrophages in biological functions and sometimes can still be replaced by recruited macrophages, especially when the tissue is damaged [38].

### Functions of macrophage

#### Phagocytosis and efferocytosis of macrophages

**Fig. 2:**
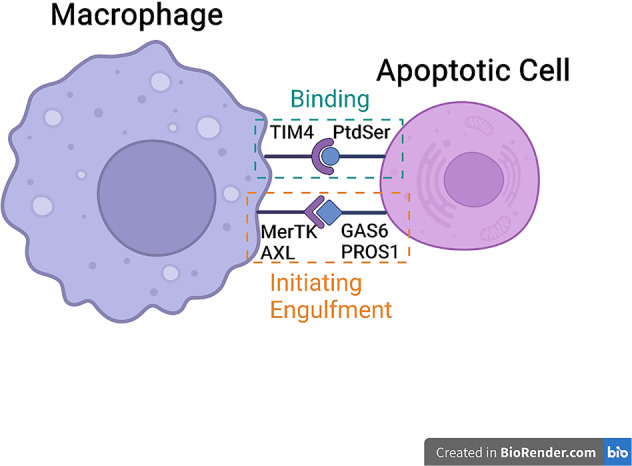
Interaction between macrophage and apoptotic cell mediates phagocytosis and efferocytosis of macrophages. As PtdSer is flipped onto the surface of dying cells, TIM4 on macrophages can identify and bind to PtdSer tightly. However, the binding cannot initiate engulfment. To engulf dying cells, TAM family, mainly MerTK and AXL, is required to bind with GAS6 or PROS1 in order to initiate rapid engulfment activity.

As a critical part in immune system, macrophages help remove the debris and apoptotic cells from the body to maintain homeostasis. Experiments suggested that endocrine-resident macrophages were more efficient in efferocytosis and phagocytosis, both in vitro and in vivo, than exocrine-resident macrophages, which may be one of the factors to induce endocrine macrophages’ polarization [71]. Efferocytosis means the remove of cells experiencing programmed cell death before further necrosis or the release of inflammation-inducing ingredients. Abnormal efferocytosis has something to do with various diseases, containing cardiovascular diseases, autoimmune diseases, brain diseases, etc. [72–74]. Interaction between macrophage and apoptotic cell is required for phagocytosis and efferocytosis of macrophages (Fig. 2). Macrophages identify apoptotic cells through the phosphatidylserine (PtdSer) on the surface of dying cells, which is known as the “eat-me” signal [75]. As PtdSer flips over to cell surface, PtdSer receptor cell immunoglobulin mucin receptor 4 (TIM4) and TAM family receptor tyrosine kinase receptors (mainly MerTK and AXL) expressed by macrophages will recognize the signal [76]. TIM4 can bind to PtdSer on apoptotic cells tightly but it fails to engulf them. It needs to cooperate with MerTK which requires growth arrest-specific 6 (GAS6) or protein S 1 (PROS1) to assist binding with PtdSer and initiates rapid engulfment activity [77, 78]. Experiments imply that substances like Angiotensin II impairs macrophages efferocytosis through AT1R/ROS/p38/MAPK/ADAM17 pathway [79]. Besides the role of MerTK in efferocytosis, the level of AXL expression can also greatly influence macrophages’ functions. The transcription factor MafB is proved to enhance the efferocytosis in RAW264.7 macrophages by upregulating the AXL expression, though its detailed mechanism is still unknown [80].

#### Cytokines secretion and polarization of macrophages

**Fig. 3:**
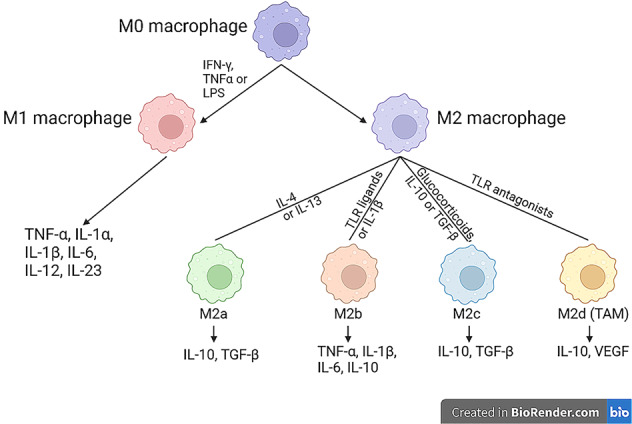
Polarization and categories of macrophage. Macrophages can polarize into two main phenotypes, M1 and M2, when stimulated by different substances. M2 macrophages can be further divided into four subtypes (M2a, M2b, M2c and M2d) for their characteristic secretion and functions in certain environment.

Macrophages are characterized by their diversity and plasticity for their rapid response to the stimuli in certain environment and convert into a specific functional phenotype, which is a process called polarization. Due to the different gene expression in different functional profiles, the secretion of cytokines is not the same in diverse states of macrophages (Fig. 3). Therefore, according to the difference in cytokine secretion and metabolic adaptions, macrophages are divided into 2 types, M1 type (classically activated or pro-inflammatory macrophages) and M2 type (alternatively activated or anti-inflammatory macrophages).

M1: M1 macrophages are recognized by their functions of killing bacteria, cleaning cancer cells and presenting antigen information to Th1 lymphocytes. M1 cells can be classically activated by IFN-γ, TNFα or LPS, and release the pro-inflammatory factors like TNF-α, IL-1α, IL-1β, IL-6, IL-12 and IL-23.

M2: M2 type macrophages can be further classified into four groups mainly according to their different stimuli, cell expression markers, secreted mediators and functions.

M2a: M2a macrophages generally aim at anti-inflammatory activity, responsible for promoting wound healing (tissue repairing) and resolution of inflammation. Induced by cytokines IL-4 or IL-13, M2a macrophages appear and mainly secret anti-inflammatory factors IL-10, TGF-β.

M2b: M2b macrophages are formed through the stimulation by immune complexes, TLR ligands or IL-1β. By producing cytokines like TNF-α, IL-1β, IL-6 and IL-10, these macrophages are mainly responsible for Th2 activation and regulation of immune reactions.

M2c: Macrophages stimulated by Glucocorticoids, IL-10 or TGF-β can be differentiated into M2c type. They mainly produce cytokines such as IL-10, TGF-β which are anti-inflammatory cytokines. Besides, these cells own the function of phagocytosis of apoptotic cells.

M2d (TAM): M2d type macrophages are quite different from other types for they exist in tumor microenvironment. These macrophages are also called tumor-associated macrophages (TAM) and they can help enhance angiogenesis and tumor growth. The stimuli of TAM are mainly TLR antagonists and they can produce cytokines like IL-10 and vascular endothelial growth factor, thus facilitating the development and metastasis of tumor [39, 40].

#### Production of reactive oxygen species (ROS)

**Fig. 4:**
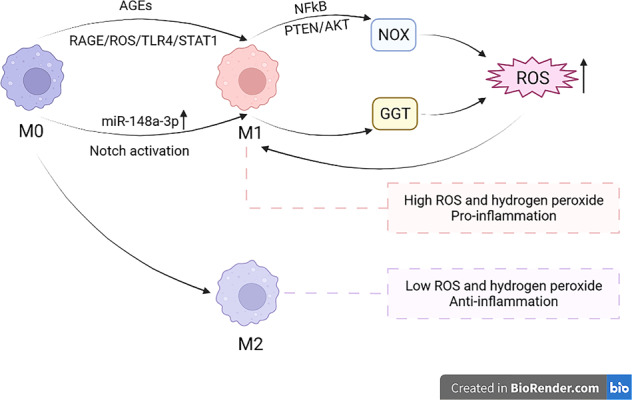
ROS is highly produced by M1 macrophages and associated with M1 polarization. After polarization of macrophages into M1 type, ROS is highly produced through two different ways, NOX and GGT, thus leading to pro-inflammation property. Besides, high level of ROS can also promote macrophages’ polarization toward M1. In contrary, M2 macrophages have low ROS and anti-inflammation property.

Virus can activate Toll like receptor 7 (TLR7) on macrophages and increase the ROS level via the nicotinamide adenine dinucleotide phosphate (NADPH) oxidase (NOX), composed of various cytosolic and membrane subunits [81]. NOX is a major enzymatic source for the production of cellular ROS under various pathologic conditions that involve synthesis of superoxide. Among all, NOX2 mediated by NF-kB pathway contributes most to the NADPH-dependent generation of ROS [82]. Besides, NOX1 and NOX4 are also found being related to the production of ROS [83]. Generally, ROS can be produced by M1 macrophages via NOX2 pathway to help kill the pathogens in the immunologic process. Advanced glycation end products (AGEs), produced by some nonenzymatic glycation reactions, can also induce the generation of ROS in macrophages and their polarization toward M1 type via RAGE/ROS/TLR4/STAT1 pathway [84]. Moreover, miR-148a-3p promoted M1 polarization of macrophages upon Notch activation and its overexpression reinforce ability to engulf and kill bacteria through excessive production of ROS. ROS is highly produced by M1 macrophages and associated with M1 polarization (Fig. 4). Further studies show that the generation of ROS is mainly due to PTEN/AKT pathway with the activation of NF-κB signaling [85]. In addition to H_2_O_2_ generated from NOX, another source of ROS in macrophages is identified as γ-glutamyltransferase (GGT), which is found in exposure to crocidolite and neuroinflammation. The oxidative injury and clinical signs in neuroinflammation can be suppressed by targeting GGT activity [86, 87]. Compared with M1 macrophages, M2 macrophages have lower levels of ROS and hydrogen peroxide, which implies M1 types’ pro-inflammatory and M2 types’ anti-inflammatory functions [88] (Fig. 4). Proper ROS assists immune protection, but excessive production of ROS will impair cell membrane and DNA, leading to cell death. Overproduction of ROS under inflammatory condition can polarize macrophages toward M1 type and decreasing ROS via NAC can partially reverses the M1/M2 polarization.

## Interaction between ferroptosis and macrophages

As is integrated above, the properties of ferroptosis and functions of macrophages have a lot in common, indicating the relationship between ferroptosis and macrophages. Firstly, iron accumulation in ferroptosis and rich iron storage in macrophages, especially in M1 type, may have something to do with each other. Secondly, in ferroptotic cells, various kinds of inflammatory cytokines are tested in experiments and the levels of them are witnessed obvious escalation. As is known to all, one of the most important characteristics of M1 macrophages is to release inflammatory factors and initiate inflammation, suggesting their interaction. Last but not least, the main cause of ferroptosis is the ROS from Fenton Reaction or depletion of GPX4 and the catalysis of lipoxidase. Studies have proved that macrophages can produce ROS to kill foreign substances like bacteria and microorganisms and the cytokines from macrophages can regulate the activity of LOX in cells, which means macrophages can participate in iron and lipid metabolism to induce ferroptosis. All these similarities have implied the link between ferroptosis and macrophages, which greatly arouse researchers’ interests.

### The effect of ferroptosis on macrophages

#### Iron metabolism affects macrophages’ polarization

**Fig. 5:**
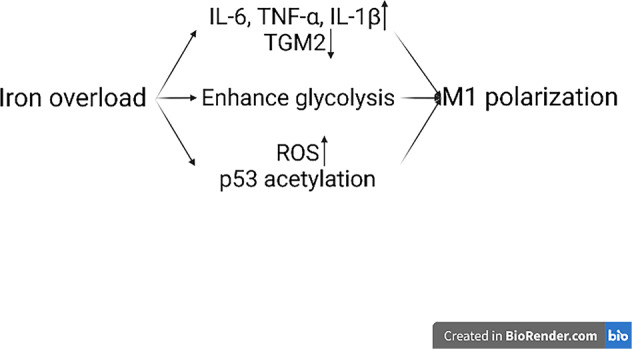
Iron overload induces M1 polarization. Iron accumulation in macrophages promotes M1 polarization in three main ways. M1 markers such as IL-6, TNF-α and IL-1β increase and M2 markers like TGM2 decrease. Iron overload can enhance glycolysis and cause M1 type. Besides, the rise of ROS level and p53 acetylation can also lead to M1 macrophages.

Iron is a significant element that participate in activities like cell proliferation, metabolism and differentiation. Most of the iron needed for physiological processes is from the recycling iron in RBCs by macrophages through complex pathways. Iron can determine macrophages’ fate and function, especially cell development and differentiation. Macrophages generally store iron by binding it to ferritin (Ft). According to different stages of macrophages polarization, the expression of Fe-related genes will shift. Compared with M2 macrophages, M1 type expresses higher Hamp and FTH/FTL, but lower FPN and IRP1/2, indicating more iron storage [89]. Iron overload induces M1 polarization (Fig. 5). Studies prove that iron overload can increase the level of M1 markers such as IL-6, TNF-α and IL-1β and decrease M2 makers like TGM2, in another word, promoting polarization to M1 macrophages [90]. In addition to inducing inflammatory factors, iron overload is found to aggravate the development of atherosclerosis by enhancing glycolysis in order to prompt M1 phenotype [91]. Besides, ROS production and p53 acetylation caused by iron overload also contribute to M1 polarization [92]. However, iron overload doesn’t always cause M1 polarization. Research in 2020 reveals that under the circumstances of chronic iron overload, THP-1 monocyte-derived macrophages tend to exhibit signs of M2 type and downregulate the markers of M1 macrophages [93].

#### Ferroptotic cells initiate macrophages’ recruitment

**Fig. 6:**
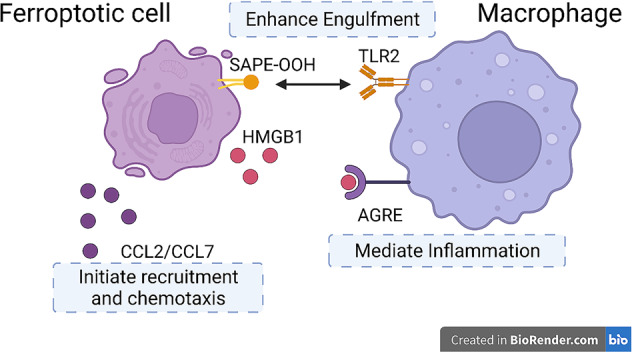
Ferroptotic cells activate macrophages’ functions and recruitment. In dealing with ferroptotic cells, TLR2 on macrophages identifies and binds to SAPE-OOH on the surface of ferroptotic cells to enhance engulfment. HMGB1 released by dying cells can interact with AGRE on macrophages in order to mediate inflammation reactions in macrophages. Some molecules like CCL2 or CCL7 can initiate the recruitment and chemotaxis of macrophages for intensity of immune reactions.

Ferroptosis is identified in many kinds of diseases, and the clearance of the ferroptotic cells is executed by macrophages. As is mentioned above, macrophages’ phagocytosis plays an important part in immune system. Ferroptotic cells activate macrophages’ functions and recruitment (Fig. 6). Damage-associated molecular pattern molecules (DAMPs) are endogenous danger signals that can recruit and activate macrophages, alerting the immune defense. In the occurrence of ferroptosis, studies suggest that ferroptotic cells can release a DAMP called HMGB1, which depends on autophagy manner. Advanced glycosylation end-product specific receptor is required for HMGB1 to mediate inflammation in macrophages [94]. In terms of dealing with ferroptosis, TLR2 on macrophages first interact with oxidized PL, 1-steaoryl-2-15-HpETE-sn-glycero-3-phosphatidylethanolamine (SAPE-OOH) on the surface of ferroptotic cells, which help improve the efficiency for macrophages to engulf ferroptotic cells. What’s more, anti-HMGB1 neutralizing antibodies or the depletion of AGRE relieves the inflammatory response in macrophages, implying that limit the expression of HMGB1 can be a method to deal with the inflammation in macrophages [95]. Besides HMGB1, ferroptotic cells triggered inflammation and recruitment of macrophages through the activation of molecule inflammatory pathways. The expression of various inflammation-related genes is induced in ferroptosis, especially CCL2 and CCL7 which assist recruitment and chemotaxis of macrophages [96–98].

### The role of macrophages in ferroptosis

#### Phagocytosis and iron accumulation

**Fig. 7:**
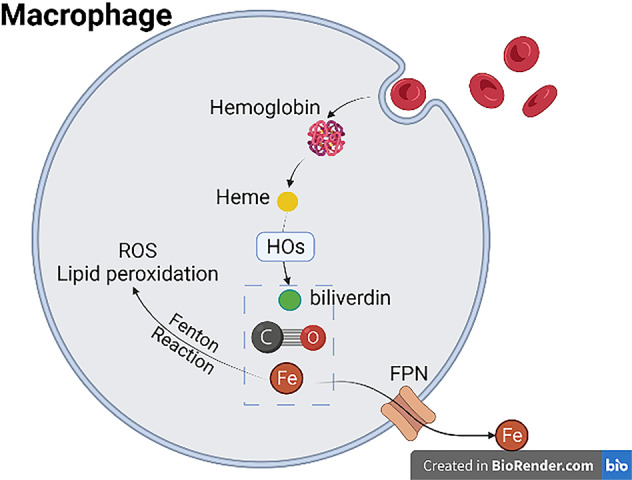
Macrophages digest RBCs and raise iron level. Macrophages engulf RBCs and digest them into hemoglobin, which further break down into heme. Heme can be decomposed into biliverdin, carbon monoxide and iron. Iron generated from heme can either promote ROS production and lipid peroxidation or released into the environment through FPN, thus raising the iron level in both macrophages and the tissue environment.

Under normal circumstances, macrophages play an important part in the recycling of iron through the engulfment of red blood cells (RBCs). As the number of RBCs suddenly rises or the RBCs are seriously damaged, increased erythrophagocytosis will happen [58] (Fig. 7). For one thing, a large number of RBCs will be eaten and digested with the help of Nramp1 in macrophages [27]. Iron overload in macrophages from RBCs is haem-iron. Haem in macrophages is first catabolized by haem oxygenases, generating iron, carbon monoxide and biliverdin [28, 29]. When it comes to resident macrophages, such as Kupffer Cells (KCs) in liver, they degrade hemoglobin into hemes and increase the iron in cells [30]. The iron accumulates in the macrophages, accompanied by the release of ROS and the generation of lipid peroxidation via Fenton Reaction, leading to ferroptosis of macrophages. For another, the iron accumulation in macrophages, especially resident macrophages, can affect other cells in certain tissue. Experiments prove that ferroportin1 in hepatocytes and macrophages are responsible for the iron mobilization between cells and bloodstream in order to store iron for the whole body [31]. Body iron content universally remain at stable levels via the hepcidin/ferroportin regulatory system. If the balance is broken by the accumulation of iron in macrophages, the unregulated iron export will cause tissue and even systematic iron overload, which creates opportunities for the occurrence of ferroptosis in tissue cells.

#### Cytokines and ferroptosis regulation

Macrophages universally execute their functions and regulate biochemical reactions through the secretion of certain cytokines. So far, some cytokines mostly produced by macrophages are reported to help induce or inhibit the process of ferroptosis via different ways.

Typically, IL-6, a marker of M1 macrophages, is found promoting lipid peroxidation and disrupting iron homeostasis in bronchial epithelial cells, thus leading to ferroptosis [41, 42]. IL-6 affect iron levels in cells by controlling the transcription of hepcidin which is a short cysteine-rich peptide hormone that can influence intestinal absorption and the release of iron in macrophages Fe stores. The transcription of hepcidin is mediated by IL-6 via the activation of JAK-STAT3 pathway [43, 44], and the expression of hepcidin is found to be activated by BMP/SMAD pathway [45]. Hepcidin first binds to ferroportin (FPN) and cause internalization of hepcidin-FPN that is soon degraded into lysosomes [46]. As a result, promoting the production of hepcidin will obviously reduce the expression of FPN, thus aggravating iron deposition. Furthermore, IL-6 can even induce the polarization of macrophages toward M1 or M2 under different circumstances [40], which is related to the occurrence of ferroptosis as well.

TNF-α is another symbolic inflammatory factor released from M1 macrophages. Its functions in lipid metabolic and inflammation are worth studying in the process of ferroptosis. First, TNF-α is able to upregulate ACSL3, a key enzyme in the synthesis of acyl-CoA, which means TNF-α promotes the lipid accumulation in cells, creating the conditions for the occurrence of inflammation response and ferroptosis [57].

As for IL-1β, studies show that it can increase the expression of FPN in glial cells through the activation of p38-MAPK pathway, which may cause the excessive iron efflux and deposition in nerve cells and the environment [47]. Besides, IL-1β secreted by macrophages is proved to upregulate the transcription of hepcidin via enhancing the expression of CCAAT enhancer-binding protein (C/EBP) [48, 49] and the expression of hepcidin with the help of phosphorylated c-Jun N-terminal kinase and its substrates c-Jun and JunB [50], thus leading to FPN degradation and iron overload. In some aspects, IL-1β and IL-6 can cause the same effect in the activation of hepcidin but through different signaling ways [51]. In fact, IL-1β is also reported to be relative to the lipid homeostasis in certain tissue, especially kidney. IL-1β urge glomerular mesangial cells (HMCs) to express lectin-like Ox-LDL receptor 1 (LOX-1) and uptake more oxidized low-density lipoprotein (Ox-LDL) [52, 53]. Therefore, the lipid accumulation caused by excessive IL-1β may easily arouse the inflammation reaction in cells and tissue.

iNOS is another typical inflammatory factor in our body. Though it is considered to promote inflammation, experiments found that it had protective functions in ferroptosis. The activation of NOX and iNOS can cause the production of ROS and RNS, resulting in the depletion of GSH and GPX and aggravating lipid peroxidation [54]. Inhibition of iNOS can reverse the ferroptosis caused by overexpression of iNOS [55]. However, recently some studies found that there is something different with the inflammatory cytokine iNOS. A study found that inhibition of iNOS by using Nω-nitro-L-arginine will make the matter worse in beta-cell death. It led to a massive lipid peroxidation in exposure to pro-inflammatory cytokines, indicating that nitric oxide help prevent the induction of ferroptosis [56].

#### ROS release and ferroptosis

ROS generated from macrophages first affect their recruitment and polarization. In fact, the role of ROS in polarization is quite complex. Under different conditions, ROS may have reversed effect on macrophages phenotype. In most cases, such as Periodontitis, diabetes, etc, ROS produced in macrophages will induce macrophages polarize toward M1 type, thus increasing the release of a series of inflammatory factors and aggravating inflammation, which provide opportunities and environment for ferroptosis [32, 33]. However, in non-small cell lung cancer, ROS from NADPH oxidase 4 is responsible for both the inflammation and M2 polarization and recruitment of tumor-associated macrophages (TAMs) via ROS/PI3K [34]. Therefore, repolarization of TAMs toward M1 to induce ferroptosis becomes the target of therapy. In addition to NADPH oxidase, mitochondrial ROS (mtROS) can be generated from the activation of TLR pathway that interacts with tumor necrosis factor receptor-associated factor 6 (TRAF6) engaging the ubiquitination of evolutionarily conserved signaling intermediate in Toll pathways. mtROS is also reported to help execute macrophages’ biological functions [35].

ROS induces ferroptosis mainly through lipid hydroperoxides or the depletion of antioxidants such as GSH or GPX4 in amino acid metabolism, which has been mentioned above in detail. For macrophages are the center of iron and lipid metabolism, related proteins in macrophages are quite active and they are linked with the generation and depletion of ROS to maintain the balance.

When it comes to iron metabolism, increased level of mtROS, usually accompanied by the decline of the expression of iron-sulfur cluster assembly scaffold protein ISCU, the antioxidant enzymes SOD1 and SOD2 and other related mitochondrial respiration components, thus affecting iron metabolism and oxidation reactions [36]. In brain, NO• acts on cytosolic aconitase (c-aconitase) and changes it into iron regulatory protein 1 (IRP1), which damages iron homeostasis through IRP1’s binding with to iron response elements in mRNAs of iron-related proteins [59].

However, ROS in cells is recently found to have reversed effects on lipid metabolism in the occurrence of ferroptosis. After discovering that NO•-affecting 15-hydroperoxy-eicosa-tetra-enoyl-phosphatidylethanolamine produced by 15-lipoxygenase (15-LOX) modulate the ferroptosis endurance in M1 macrophages [60], researchers further found that ROS like O2 and NO• uses the same entry pores and channels connecting to 15-LOX-2 catalytic site for competition [61]. Besides, since NO• is small enough to go through the membrane, NO• produced by macrophages can even suppress PA stimulated ferroptosis in distant epithelial cells [62].

## Cancer treatment by targeting macrophages

Experiments have proved that M2-like tumor- associated macrophages infiltration is the promotor of the development of cancer. Cancer cells in liver are able to interact with M2 macrophages via Wnt/β-catenin signaling and trigger M2 polarization in macrophages, reinforcing tumor activities [63, 64]. In terms of the key role of macrophages in ferroptosis, researchers start to find new ways to treat cancer by targeting macrophages and inducing ferroptosis. Newly published articles concerning tumor therapies on the basis of macrophages are mainly aims at the repolarization, changing M2 type TAM to M1 type. Engineered magnetosomes delivered to the tumor tissue are reported to induce macrophages’ polarization from M2 toward M1 and release more Fe ion, thus producing excessive hydrogen peroxide and impelling ferroptosis [65]. Other reports indicate that zero-valent-iron nanoparticle (ZVI-NP) can activate repolarization and downregulate the population of regulatory T cells to initiate the anti-tumor immune activity [66]. As a ferroptosis-promoting agent, Herein switches mitochondrial oxidative phosphorylation to glycolysis in TAM cells and induces ferroptosis stress, resisting anti-inflammatory reactions and promoting pro-inflammatory signaling pathways [67]. In addition to inhibit primary tumor, this agent can provoke immune response against cancer through binding with an immune checkpoint blockade. Stimulated phagocytes and enhanced tumor antigens uptake assist immunotherapy with few abnormalities [68]. In lung adenocarcinoma patients, RRM2 inhibition promotes M1 polarization and suppresses M2 polarization, which can be reversed by ferroptosis inhibitors [69].

However, some researchers prove that treating cancer by inducing ferroptosis may have shortcomings. They discover that in pancreatic ductal adenocarcinoma, ferroptosis can result in experimental pancreatitis and assist Kras-driven pancreatic tumorigenesis, which should be taken into account before applying ferroptosis for treatment [70].

## Prospect

So far, ferroptosis has been found to have an impact on the development of a variety of diseases, thus targeting the regulation of ferroptosis process may be a new therapy. However, most of the experiments mainly aim at inducing or inhibiting ferroptosis in tissue cells directly. Few articles use the relationship between macrophages and ferroptosis to deal with diseases. Therefore, this review is expected to provide inspirations for future studies.

In terms of liver damage, targeting ferroptosis is found to have an effect on relieving or aggravating the symptoms, indicating the role of iron, lipid and amino acid metabolisms. Non-alcoholic fatty liver disease (NAFLD), like non-alcoholic steatohepatitis (NASH), can be promoted by iron accumulation, lipid peroxidation, GPX4 depletion and inflammatory initiation, which are main processes of ferroptosis [99–101]. After given ferroptosis inhibitors, such as liproxstatin-1 or ferrostatin-1, NASH is greatly alleviated [102, 103]. KCs, the residents macrophages in liver, are found to have enhanced phagocytic dysfunction and closely related to iron homeostasis, thus influencing ferroptosis in the development of NAFLD [104–107]. Besides, KCs participate in the intake and clearance of lipid through M1 type polarization with the help of invariant natural killer T cells in NASH, indicating the role of KCs in lipid metabolism [108–110]. As the studies above imply, the regulation of KCs can influence the development of ferroptosis especially in iron and lipid metabolisms, providing a new possible therapy for NAFLD.

Abundant evidence has proved the link between ferroptosis and neurological diseases, like Alzheimer’s disease, Parkinson’s disease, Huntington’s disease and other brain injury [111–115], accompanied by the occurrence of neuroinflammation. A number of macrophages in nerve tissue, which are called Hortega cells or microglia cells, are involved in the regulation of neuroinflammation. Since typical manifestations of ferroptosis and inflammation, such as iron accumulation and GPX4 depletion, are found in these diseases, researchers pay attention to ferroptosis and microglia cells in therapy for injury. After RSL3 treatment, a ferroptosis inducer, the inflammatory reactions of microglia are greatly triggered, and different types of microglia exhibit various sensitivity to ferroptosis, which is consistent with results in other researches. However, RSL3 can attenuate lipopolysaccharides-induced inflammation by targeting Nrf2 expression [116]. Some of ferroptosis inhibitors, including liproxstatin-1 and an arylthiazine backbone (ADA-409-052), are reported to protect against neurological diseases and neuroinflammation through mediating the state of microglia cells [117, 118]. Thus, targeting microglia to regulate ferroptosis in neurological disorders or brain injury is worth further studying.

In other tissues, relationship between resident macrophages and diseases or ferroptosis and diseases are respectively proved. However, the relationship between these resident macrophages and ferroptosis is still unclear. For example, ferroptosis and macrophages are respectively found participating in heart diseases, like ischemia/reperfusion, heart failure, drug induced cardiotoxicity and so on, but few research focus on the interaction between ferroptosis and heart macrophages [119–124]. If the direct relation between ferroptosis and resident macrophages can be veiled in future experiments, it will probably contribute to new therapies.
